# Case Report: Robotic ALPPS Procedure for Hepatocellular Carcinoma in the Right Lobe of the Liver

**DOI:** 10.3389/fsurg.2021.655683

**Published:** 2021-04-13

**Authors:** Evgeny Solomonov, Itamar Tzadok, Salomon Stemmer, Seema Biswas

**Affiliations:** ^1^Department of General and Hepatobiliary Surgery, Ziv Medical Center, Safed, Israel; ^2^Department of Transplantation, Rabin Medical Center and Tel Aviv University School of Medicine, Petah-Tikva, Israel; ^3^Hepatobiliary Surgery Unit, Rabin Medical Center and Tel Aviv University School of Medicine, Petah-Tikva, Israel; ^4^Institute of Oncology, Davidoff Center, Rabin Medical Center and Tel Aviv University, Petah-Tikva, Israel

**Keywords:** liver, robotic, associating liver partition and portal vein ligation for staged hepatectomy, hepatocellar carcinoma, hepato-pancreatobiliary surgery

## Abstract

**Introduction:** Associating liver partition with portal vein ligation for staged hepatectomy (ALPPS) is a surgical procedure for liver malignancy where the volume of the liver remnant is estimated to be too small. We present the first case of two-stage robotic ALPPS procedure, illustrating the steps and advantages of robotic surgery.

**Materials and Methods:** A 68-year-old man with morbid obesity (BMI 40), portal fibrosis, macrovesicular steatosis, and poor liver function underwent robotic ALPPS for hepatocellular carcinoma in the right lobe of the liver (segments 5, 7, and 8). A video presentation (https://youtu.be/M50Gumf-4pw) of the operative procedure is accompanied by explanation in the text with embedded corresponding video time points.

**Results:** Both stages of the procedure were performed robotically, with negligible blood loss, and rapid surgical recovery. The patient died 3 years later.

**Discussion:** Robotic ALPPS offers reduced morbidity in major liver surgery for malignancy and may extend survival in meticulously selected patients.

## Introduction

Associating liver partition with portal vein ligation for staged hepatectomy (ALPPS) is a new procedure for the surgical management of malignancy of the liver where the volume of the liver remnant is estimated to be too small. It is a variant of the 2-stage hepatectomy, inducing rapid and marked hypertrophy of the future liver remnant (FLR) through the fundamental auxiliary role assumed by the deportalized liver after the first critical postoperative week. This potentially curative surgery is associated with a lower risk of postoperative liver failure (PLF) (1, 2). The ALPPS procedure has been successfully performed using open and laparoscopic approaches (3–7). The video accompanying this article shows the first totally robotic ALPPS procedure performed in the world (July 2013) and was formally presented at the Worldwide Congress of the 5th Clinical Robotic Surgery Association (CRSA) in Washington DC, in October 2013. The patient had hepatocellular carcinoma in the right lobe of the liver (segments 5, 7, and 8). Vicente and colleagues have since published their experience (in 2016) of totally robotic ALPPS in a patient with colorectal liver metastases in liver segments 1 and 4 (8). Krishnamurthy et al. reported 1-stage robotic ALPPS in India in 2018 (9). Evidence is emerging for the advantage of APPPS over portal vein embolization (PVE) in achieving greater hypertrophy of the future liver remnant sooner (10–14). This is an important factor in patients who already have impaired liver function.

## Materials and Methods

We present the first case of ALPPS procedure using a totally robotic approach for both steps of surgery in Israel in 2013. Robotic surgery is now well-established in centers around the world, especially for urological, colorectal, and gynecological procedures (15). Fourth generation Da Vinci robots were introduced in 2014—the Da Vinci Xi® Surgical System robot (Intuitive Surgical, Inc., Sunnyvale, California). We used the newest at that time in 2013—the Da Vinci Si® Surgical System robot (Intuitive Surgical, Inc., Sunnyvale, California), combining a three-dimensional high-definition vision system (two cameras in one) which magnifies images, a control console, and 3 or 4 robotic arms through which EndoWrist® instruments may be manipulated, through which a camera, controlled by the surgeon, is inserted, and for retraction. The range of robotic surgical indications, initially, urological—an innovation in precision nerve preserving prostatectomy with theoretically improved sexual function and urinary continence—has since extended use of the technique to gynecological, colorectal, cardiac, thoracic, and even head and neck surgery. Recent advances in liver resection have focused on the advantages of robotic surgery in segmental or sub-segmental resection over laparoscopic lobar resection, and robotic access to difficult-to-reach lesions that may be locally excised avoiding lobar resection with the additional morbidity this entails (16).

There are notable advantages of robotic surgery over traditional laparoscopic surgery. Stable operative images are maintained throughout the procedure by the dual high definition camera system and the Da Vinci computer. This permits surgery under magnification, effectively rendering the camera an intraoperative microscope, and eliminating the need for surgeons to wear intraoperative loop magnifying glasses. The second advantage lies in EndoWrist® instrumentation. EndoWrist® instrumentation comprises what is effectively a system of small joints, simulating but exceeding the range of movement of the human wrist and, in terms of operative dexterity, permitting fine movements more in keeping with advanced open surgical techniques than the more restrictive movements of laparoscopic instruments. This enhanced fluid ambidexterity, precision, intuitive motion, and finger-tip control, with 7 degrees of freedom, combined with motion scaling and tremor reduction (both physiological and associated with prolonged surgery), translates the movements of the surgeon's wrists, hands, and fingers to perform precise, real-time movements of surgical instruments. Thus, the system provides maximal responsiveness and facilitates rapid and precise suturing, dissection, and tissue manipulation in a minimally invasive procedure within the confines of the abdominal cavity (17). A further advantage is that the Da Vinci System effectively gives the surgeon an extra pair of hands. The surgeon sitting at the console moves two joysticks with different foot pedal combinations that reproduce the work of four arms. One of these arms holds the camera while the three others hold working surgical instruments. This design allows relative independence of the surgeon from his assistant and prevents misunderstanding in camera movement, substantially improving the stability of images throughout surgery.

The features described above enhance the quality of robotic over laparoscopic surgery, especially in deep, hard-to-reach, complex anatomical areas, and in dealing with the challenges incumbent in liver and pancreas surgery (resection of the caudate lobe or centrally placed tumors, and bile duct resection and anastomosis, for example). Robotic surgery has, therefore, increased the scope of minimally invasive hepatobiliary surgery possible as the prohibitive lack of flexibility of laparoscopic instruments is overcome and the permissive fine movements necessary for careful dissection, control of bleeding, and anastomosis are permitted through the console of the robot (18). The learning curve in advanced surgical cases, however, is long. Reproducibility and the rapid expansion of laparoscopy in liver surgery has meant that robotic surgery has been met with some skepticism.

As robotic procedures increase across the world, however, it is important to emphasize the experience and expertise necessary in both advanced laparoscopy and complex hepatobiliary surgery to competently perform robotic resection (19, 20). There is growing evidence that poor patient selection and a lack of training are implicated in complications arising after robotic surgery. Robotic surgery should be performed by well-trained, experienced surgeons in centers of excellence for training and outcome. The selection of patients for robotic surgery over open or laparoscopic surgery rests on the superiority of robotic surgery in the control of bleeding and oncological resection with clear margins, making for safe cancer surgery. In our center the vast majority of liver resections were performed robotically (between 2008 and 2014, when the main author was head of the HBP unit). Our 2-year experience amounted to over 60 cases of liver resection, mainly for cancer. Forty three percent of cases required major liver resection such as right or left hepatectomy, central liver resection, right extended hepatectomy, radical cholecystectomy for cancer, and liver resection combined with the radical resection of other organs.

Based on this experience we introduced robotic surgery to both stages of the already complex ALPPS procedure (with its attendant physiological assaults), in the expectation of diminishing physiological trauma, bleeding, and operative morbidity. The video demonstrates the world's first case of ALPPS in a morbidly obese patient (BMI 40) with diseased liver parenchyma (fatty liver and liver fibrosis), reduced preoperative liver function, and hepatocellular carcinoma (i.e., not meeting the Milan criteria for liver transplantation) undergoing resection of the right lobe of the liver.

### Case Description: (https://youtu.be/M50Gumf-4pw)

A 68-year-old man was referred to our clinic for the evaluation of elevated serum liver enzymes (ALT and AST levels were 1.5 times the upper limit of normal with normal GGT and ALP over the last 10 years). Bilirubin, albumin, and INR levels were normal. His previous medical history included diabetes mellitus, hypertension and morbid obesity with a BMI 40. Viral and autoimmune markers for liver disease were negative. Ultrasound scan showed severe fatty infiltration of the liver. A diagnosis of non-alcoholic fatty liver disease (NAFLD) was made. A further ultrasound scan demonstrated a 7 cm hypoechogenic mass in the right lobe of the liver (segments 5, 7, and 8). Computed tomography (CT) scan confirmed the diagnosis of a liver mass with features strongly suggestive of hepatocellular carcinoma (00:20-00:37). There were no clinical or radiographic signs of cirrhosis. The alpha fetoprotein level was normal (2.2 ng/ml). Liver biopsy from the mass and the surrounding parenchyma was performed and histopathological examination revealed the mass to be hepatocellular carcinoma, while the parenchyma had features of mild portal fibrosis and moderate macrovesicular steatosis.

The consensus in the multidisciplinary team conference was that right liver lobectomy should be performed if possible. The concerns were combined high risk of postoperative liver failure because of pre-existing liver disease and poor liver function, and operative morbidity associated with morbid obesity. These factors favored a 2-week staged ALPPS over the more prolonged stages of PVE. The decision was taken to perform robotic ALPPS with the intention to reduce these risks.

### Video Description

#### First-Stage Surgery: ALPPS (8 July 2013)

Under general anesthesia, a laparotomy was performed through a small Pfannenstiel incision. The GelPOINT access platform (Applied Medical Ltd) and pneumoperitoneum were applied. An 11 mm trocar was inserted 3 cm above the horizontal transumbilical line and in between the right midclavicular and right parasternal lines. This trocar was used for the laparoscopic robotic camera. Three 8 mm trocars were then inserted in the subcostal areas: the first, in the right anterior axillary line, ~3 cm above the camera trocar; the second, in the left midclavicular line at the same level as the first; and, the third 6 cm lateral and 3 cm above the second trocar. The Robotic Surgical Da Vinci Si System was connected to the trocars. Finally, an assistant 12 mm trocar was inserted through the right low abdominal wall to use mainly for suction, provide extra tissue traction and insert sutures into the surgical field as needed.

Intraoperative ultrasound (00:44) confirmed the preoperative diagnosis. Upper abdominal exploration was performed and both liver lobes were mobilized through division of the triangular, falciform and coronary ligaments of the liver. Partial hepato-caval mobilization was performed (00:40-00:55) at the beginning of the surgery because of a very thin caudate lobe and the remarkably enlarged and fatty right liver lobe encountered. This required retraction-elevation in order to achieve safe manipulation on the surface of the inferior vena cava and to control short hepatic Spigelian veins (00:55-01:31). It was decided that this step be completed after complete liver parenchymal transection.

The video shows cholecystectomy and ligation of the cystic duct (02:10-02:37). The hepatoduodenal portal triad structures (common hepatic artery, portal vein, common bile duct) were secured within a yellow vessel loop (02:42-02:55) as in the Pringle maneuver in order to minimalize bleeding while the gallbladder was used as a “handle” to facilitate the mobilization of the liver and visualization of the major vessels. We proceeded to hilar dissection and exposure of the right portal vein and right hepatic artery and duct (02:55-03:20). The right portal vein was tied, sutured (03:56-05:29) and divided (05:54-06:01), leaving the right lobe with arterial blood supply only. The right hepatic artery and the right hepatic duct were then marked with long colored vessel loops—red (03:20-03:56) and yellow (06:13-06:36), respectively. This marking facilitated the identification of those vessels during the second stage procedure.

Complete parenchymal liver transection was achieved, revealing the anterior wall of the inferior vena cava (06:02-06:35). The caudate lobe was then safely and completely transected (06:50). Only the right hepatic vein (07:37) was left to permit adequate right lobar venous drainage. The gallbladder was the only resected specimen at this stage and was removed from the abdominal cavity within an Endobag™ (Applied Medical Ltd) (06:35-06:48). After meticulous hemostasis the right lobe of the liver was wrapped in a nylon sheet (07:44-07:57) in order to prevent adhesions to surrounding tissues until the second stage of surgical treatment. Two Jackson Pratt drains were left between the lobes of the liver and the right diaphragm in order to prevent fluid collections in the area (07:57-08:05).

The duration of first stage surgery was 410 min. Blood loss was ~500 ml. No blood products were transfused during surgery. The patient was transferred to the recovery room and ventilated for the first 12 postoperative hours. The patient remained stable with no evidence of bleeding or hypothermia. Urine output was within normal limits and the patient was successfully extubated. The patient returned to the ward 24 h after first stage surgery. On the ward, his postoperative recovery was uneventful, and the patient was able to walk independently by the 3rd postoperative day. By the 4th day he was able to eat a normal diet. Liver function tests returned to normal limits by the 7th postoperative day. On this day he underwent three-phase liver computer tomography with volumetry of the left lobe of the liver. Compared with preoperative liver volume, more than 30% left lobe enlargement was observed (08:16-08:27). The patient was discharged home.

#### Second Stage Surgery: Robotic Completion of Right Liver Lobectomy (22 July 2013)

One week later, the patient was readmitted electively for second stage surgery—robotic completion of right liver lobectomy. Surgery was performed 14 days after initial ALPPS.

Under general anesthesia, the Pfannenstiel incision was re-opened and GelPOINT access platform reinserted. A pneumoperitoneum was applied, trocars inserted and the Da Vinci Robotic Surgical System assembled as before. A severe inflammatory reaction was observed around the wrapped right liver lobe with a thin layer of fibrin covering the nylon sheet which required slow and gentle removal in order to identify the colored vessel loops marking the right hepatic artery, right hepatic duct and right hepatic vein (08:30-08:53). The left lobe of the liver was remarkably hypertrophied. The nylon sheets were removed within an Endobag™ through the GelPOINT access platform. The right hepatic artery and the duct, and right hepatic vein were stapled and transected using a 45 mm Endo GIA purple cartilage stapler (Covidien Ltd) (08:53-09:21) and the right lobe of the liver was detached, inserted into a large Endobag™ (Applied Medical Ltd) and removed from the abdomen (09:36-09:45).

The left lobe of the liver was carefully inspected for bleeding points or bile leaks. A Jackson Pratt drain was left in the right liver bed. The Robotic System was undocked, and the specimen removed from the abdominal cavity through the Pfannenstiel incision. All surgical wounds were then sutured and infiltrated with Bupivacaine local anesthesia. The duration of the second stage was 180 min. Blood loss was ~500 ml and no blood products were transfused. The patient was extubated at the end of the procedure and observed in the Recovery Room over the next 6 h. He was transferred to the ward stable, conscious, and with a good urine output.

## Results

No opioid analgesia was required after the first postoperative day of either stage of surgery. The postoperative recovery was smooth, and he was discharged from hospital on the third postoperative day with virtually normal liver function tests. The patient recovered quickly and returned to his normal physical activity within 2 weeks. At 3-month follow up, he was well, with normal liver function (INR = 1.1), normal alpha fetoprotein levels and with no pathological lesions within a well-hypertrophied liver remnant (the left lobe). Table 1 shows 3-month follow-up data.

**Table 1:** Pre- and postoperative follow-up data.

	**Before surgery**	**1 day after 1st stage**	**1 day before 2nd stage**	**1 day after 2nd stage**	**3 months after 2nd stage**
ALT (U/L)	51	1,050	50	52	26
AST (U/L)	47	955	35	47	37
ALK phosphatase (U/L)	63	45	166	122	103
GGT (U/L)	71	59	216	152	122
Albumin (g/dl)	4.4	–	2.7	2.5	3.7
Bilirubin (mg/dl)	1.1	1.27	0.35	0.23	0.7
INR	1.1	2.2	1.2	1.9	1.0
Volumetry	^*^TLV = 3,221 ml ^†^FLR = 1,087 ml ^‡^TV = 113 ml	–	^†^FLR = 1,463 ml	–	^*^TLV = 2,805 ml

* *TLV, Total Liver Volume*;

† *FLR, Future Liver Remnant*;

‡ *TV, Tumor Volume*.

Unfortunately, a year after ALPPS surgery, the patient developed peritoneal recurrence. He responded well to a 4-month course of Sorafenib and remained stable with active peritoneal disease. He received no further chemotherapy over the next year and was subsequently enrolled in clinical trial MK 3475-240. Although, eligible at the beginning of the trial (CT scan revealing a small volume of ascites—not clinically detectable), after the first dose of study medication he developed a large volume of ascites. Six liters of ascitic fluid was initially drained, followed by a further 5 L 3 weeks later. Serum bilirubin levels remained normal and liver enzyme levels were stable at twice the level above the normal range. Thereafter, his condition deteriorated rapidly, and he died at home more than 3 years (38 month) after ALPPS procedure.

## Discussion

The robotic ALPPS procedure is a revolutionary approach to complex liver surgery. It is a minimally invasive approach to surgery in patients with considerable co-morbidity and pre-existing liver disease. Robotic laparoscopic portal vein ligation associated with *in-situ* splitting was used to induce accelerated left lobe hypertrophy in this patient. The ALPPS technique results in the detachment of interlobar portal collaterals in addition to portal vein ligation. This causes redistribution of hepatotrophic factors to the future remnant. Although the excluded liver has no portal flow, and is supplied by arterial vessels only, it acts as an auxiliary liver contributing to overall liver function until the contralateral lobe has grown sufficiently to support physiological function similar to that of a normal liver (1–3). There is growing evidence to support greater hypertrophy of the future liver remnant in ALPPS compared to PVE (10–14). Thus, as both stages of ALPPS may be achieved within 2 weeks, compared to 4–8 weeks in PVE, the advantage of ALPPS in patients with pre-existing liver disease and poor liver function is clear. As the morbidity and mortality associated with ALPPS decreases and familiarity with the surgical technique increases, a clear advantage in selected patients is emerging. As always, careful discussion within an expert multi-disciplinary team in specialist centers is essential.

The authors suggest considering this strategy in meticulously selected patients who require major hepatectomy with small remnants *per se* (<25% of total liver volume), patients who have impaired liver function due to underlying liver disease, and patients who have what we term “functionally small remnants.” The robotic approach played an extremely important role in reducing surgical morbidity and added value in terms of uneventful postoperative recovery in this high-risk patient, and in extending survival with quality of life.

Plastic bag wrapping of the liver should possibly be avoided as this resulted in a severe local inflammatory reaction against the plastic and the deposition of a large amount of sterile fibrin, which made visualization of marked structures difficult at second stage surgery.

The complexity of robotic ALPPS mandates that surgery is performed only by experienced hepatobiliary surgeons with expertise in advanced robotic and laparoscopic surgery, and that surgery is performed in specialist centers.

## Data Availability Statement

The original contributions presented in the study are included in the article/Supplementary Material, further inquiries can be directed to the corresponding author/s.

## Author Contributions

ES and SB wrote and revised the manuscript. IT assisted with figures, references, and revision. SS revised for intellectual content. All authors contributed to the article and approved the submitted version.

## Conflict of Interest

The authors declare that the research was conducted in the absence of any commercial or financial relationships that could be construed as a potential conflict of interest.
